# Predictive value of POSSUM score in surgery of acute abdomen in cirrhotic patients

**Published:** 2013-12-25

**Authors:** P Banu, F Popa, VD Constantin, C Balalau

**Affiliations:** “Carol Davila” University of Medicine and Pharmacy, Bucharest, General Surgery Department, “Sf. Pantelimon” Hospital, Bucharest

**Keywords:** audit, liver cirrhosis, acute abdomen

## Abstract

Abstract

Introduction: As liver cirrhosis has an increasing incidence in the general population and the life expectancy for these patients has increased, surgery procedures practiced for acute abdomen in such category of patients are more frequent.

Aim: To evaluate the predictive value of POSSUM score in cirrhotic patients undergoing abdominal surgery in emergency cases.

Material and method: A prospective study based on 115 consecutive patients with liver cirrhosis hospitalized and operated in the first 24 hours from admission for acute abdomen. The patients’ stratification was done by using Child Pugh score for liver cirrhosis. POSSUM score was calculated for each patient and postoperative outcomes were compared with prediction based on this score.

Statistical data analysis was made by using the chi-square test and a p value of less than 0,05 was considered to be statistically significant.

Results: There were 33 patients in stage Child A of cirrhosis, 54 in stage Child B and 28 in stage Child C. For Child A group, the POSSUM score had a satisfactory prediction in terms of morbidity and mortality. In advanced stages of liver cirrhosis, Child B and C, mortality had high rate and the observed outcomes were outside the area of POSSUM score prediction.

Conclusions: POSSUM score offers a satisfactory prediction for morbidity and mortality in emergency abdominal surgery for patients in compensated stages of liver cirrhosis. In advanced stages of cirrhosis high levels of mortality cannot be predicted by using POSSUM score.

## Introduction

Liver cirrhosis has an increasing incidence in the general population. It is estimated that, in Europe, there are 170,000 annual deaths due to this cause with major differences between regions. Thus, in the south-east cirrhosis mortality rate is 10-20 times higher than the rest of the continent [**1**].

 Romania is situated on the second place with figures of 64 males and 26,7 females considering the death rate per 100,000 population. The main causes of the disease are represented by chronic alcohol consumption, infections with hepatitis viruses B and C, and obesity related metabolic syndromes [**2**].

In these circumstances, abdominal surgery in patients with liver cirrhosis has an increasing frequency.

Liver cirrhosis is a chronic and slowly progressive disease characterized by a replacement of normal liver tissue with scars. Liver function is progressively altered leading to hepatic failure.

The effects of cirrhosis are not limited to liver function, but also affect other organs and systems. The renal, cardiac and respiratory functions are also adversely affected. These, along with coagulation disorders and immune deficiency create premises of a high-risk surgical ground [**3**].

Surgery for “acute abdomen” in such patients has a high mortality rate and requires special measures of resuscitation [**4**].

 Copeland and al. proposed, in 1991, The Physiologic and Operative Score for enUmeration of Mortality and Morbidity (POSSUM) as a scoring system for the surgical audit [**5**]. Considering both pre- and intra-operative commonly measured parameters, this score is easy to use and has a wide application for general surgery in emergency and elective procedures [**6**,**7**]. 

The aim of the present study is to evaluate the accuracy of POSSUM score in predicting outcomes after emergency abdominal surgery in patients with liver cirrhosis.

## Material and method

Our study is based on a prospective analysis between January 2008 and December 2012 on 115 consecutive patients with liver cirrhosis hospitalized and operated for acute surgical abdomen in Surgery Clinic of “Sf. Pantelimon” Hospital in Bucharest. The criteria for the patients’ inclusion in the group were the following:

 - Liver cirrhosis as a background disease

- The presence of an abdominal surgical problem whose solution was imposed in the first 24 from admission as an emergency solution

In order to assess the determinant factors of postoperative morbidity and mortality in these patients we considered the following:

- Evolutive stage of the liver disease

- Physiological status of the patient at the moment of operation

- Type and magnitude of the surgical procedure

For the staging of the liver cirrhosis we choose Child – Pugh classification. This classification is an useful instrument in emergency conditions because of its small number of parameters which can be rapidly evaluated – serum albumin level, total bilirubin, prothrombin time INR, the presence and grade of ascites and grade of encephalopathy [**8**].

 This score has proven its usefulness in many studies involving patients with liver cirrhosis, whether it was the rupture of esophageal varices, hepatocellular carcinoma, Budd-Chiari syndrome or portal subclinical encephalopathy [**9**-**12**].

 In a study conducted over a period of 12 years Mansour used Child – Pugh score as a prognosis factor for non hepatic abdominal surgery [**13**]. 

Despite the criticism that brings into question that the score is based upon components empirically chosen or that its variables are not independent predictors, studies show that the data obtained with this score have statistical significance [**14**]. 

 In light of the above, Child-Pugh score remains an instrument which is easy to use in the evaluation of the prognosis of the patient with liver cirrhosis and its predictive value is at least comparable to some elaborated scores such as MELD [**15**], elements which have imposed their use in our study.

The physiological status of patients and magnitude of surgery procedure were evaluated based on the POSSUM score.

This score quantifies twelve significant and independent physiological variables which evaluate the physiological status of the patient at the time of surgery. The patient's respiratory and cardiac function are assessed by using information that can be obtained quickly and easily by clinical and laboratory common methods - the presence and degree of dyspnea and its degree of severity, chest X-ray appearance, presence of arrhythmias on the electrocardiogram, blood pressure, pulse, the presence of angina or an underlying heart disease requiring inotropic therapy or anticoagulant. In addition to these are the laboratory tests such as hemoglobin, WBC count, blood urea nitrogen, sodium and potassium. Moreover, the patient's age and Glasgow score are also considered. The values obtained for the physiological score calculated at the time of intervention can have values between 12 and 88 (**Table 1**).

**Table 1:**
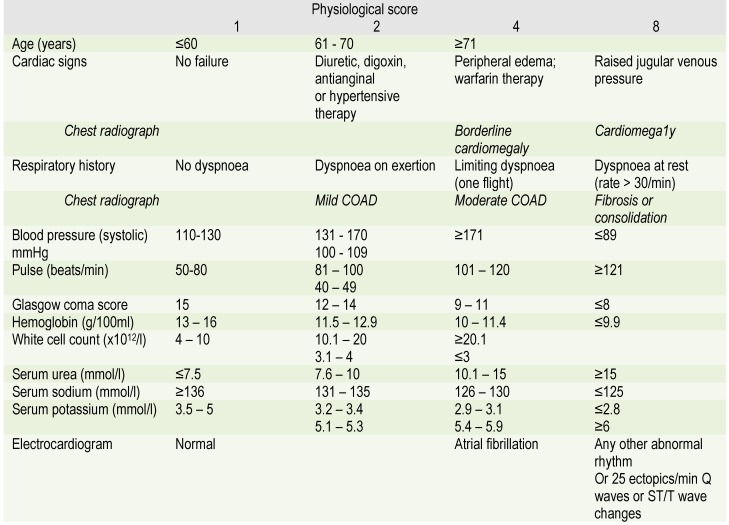
POSSUM physiological score according to Copeland and al.

Surgical trauma is evaluated based on six factors of the severity of procedure. This is classified into four categories taking into account the extent of surgery, emergency or elective nature of the operation, amount of blood lost during surgery, duration, peritoneal contamination and the presence / extension of a neoplasm (Table 2).

 Once the score is known, it is possible to estimate the risk of morbidity (R2) and mortality (R1) by using two equations. For morbidity the equation is the following:

 Log (R2 / (1-R2) = -5.91 + (O.16 X physiological score) + (0.19 X severity score of the surgical procedure)

 For mortality the equation is the following:

Log (R1 / 1 R1) = -7.04 + (0.13 X physiological score) + (0.16 X severity score of the surgical procedure).

The physiological score was assessed at the time of surgery and the operative score at the moment of the patient’s discharge.

Comparing the obtained results with those estimated by using POSSUM score, we tried an appreciation of the influence upon postoperative outcome of the physiological status and evolution stage of liver cirrhosis in these patients.

Statistical data analysis was based on X²(chi-square) test and the p value of less than 0,05 was considered statistically significant.

**Table 2:**
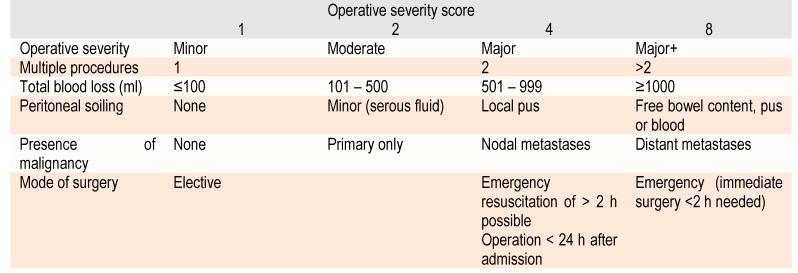
POSSUM operative severity score

## Results

Our group was composed of 115 patients, 45 females (39%) and 70 males (61%) and their ages ranged from 29 to 85 years, with an average of 60.27. 

The etiology of liver cirrhosis in patients represented in our group was especially chronic consumption of ethanol and hepatitis virus infections, groups including 102 cases - 60 and 42 patients, respectively. To these 8 cases of cardiac cirrhosis and other 5 in which the etiology could not be documented, being labeled as cryptogenic, were added.

**Fig. 1:**
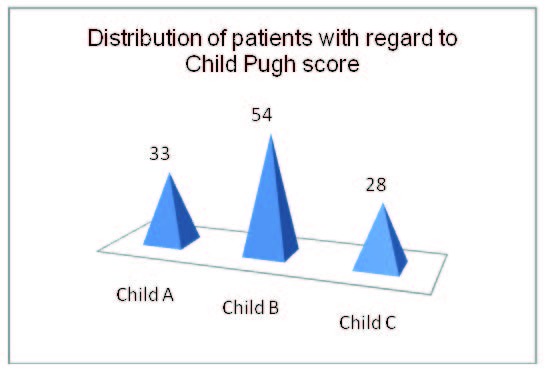
Distribution of patients with regard to Child Pugh score

The classification of patients into Child groups depending on the evolutionary stage of liver disease was the following: 33 of them (28.7%) were classified in group Child A, 54 (47%) in Child B stage and 28 (24.3%) in group Child C(**Fig. 1**).

**Fig. 2:**
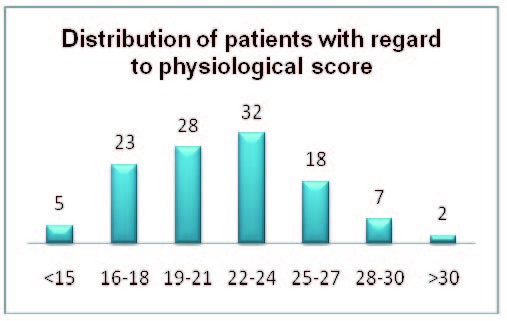
Distribution of patients with regard to physiological score

The preoperative assessment based on the physiological POSSUM score resulted in dividing the patients into the following groups score: below 15,5 cases; 23 cases with a score between 16 and 18, 28 cases with 19 to 21 points, 32 cases with 22 to 24 points, 18 cases with 25 to 27 points, 7 cases with 28 to 30 points, 2 cases over 30 points (**Fig. 2**).

The pathological conditions in our group of 115 patients who required emergent surgery were the following: 22 cases of complicated umbilical hernias, 12 complicated postoperative incision hernias, 8 complicated inguinal hernia, 35 acute cholecystitis, 14 peptic ulcer bleeding, 6 perforated ulcer, 3 penetrating abdominal wounds, 4 acute appendicitis, 2 traumatic ruptures of spleen, 2 choledocholithiasis with cholangitis, 7 bowel obstructions.

 Surgery procedures practiced in emergency conditions – in the first 24 hours from admission – on our group of patients were the following: splenectomies – 2 cases, umbilical hernia repair – 22 cases, inguinal hernia repair – 8 cases, incision hernia repair – 12 cases, suture of ulcer perforation – 3 cases, excision of ulcer – 2 cases, gastric resection – 5 cases, gastrotomy with hemostasis "in situ" for bleeding ulcer – 10 cases, resection of small intestine – 3 cases, appendectomy – 4 cases, right hemicolectomy – one case, transverse colon segmental resection – one case, Hartmann operations – 2 cases, laparoscopic cholecystectomy – 21 cases, open cholecystectomy – 14 cases, laparotomy – 3 cases, choledochotomy with Kehr drainage – 2 cases.

**Fig. 3:**
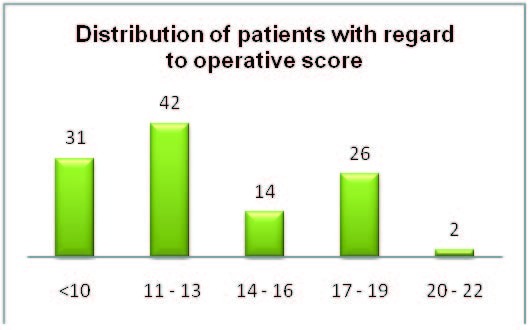
Distribution of patients with regard to operative score

 Calculating POSSUM operator score for our patients resulted in a classification into the following groups: 31 patients with a POSSUM operator score below 10, 42 patients with a score between 11 and 13; 14 patients had a score of 14 to 16; 26 cases with 17 to 19 points; 2 patients with 20 – 22 points (**Fig. 3**).

**Fig. 4:**
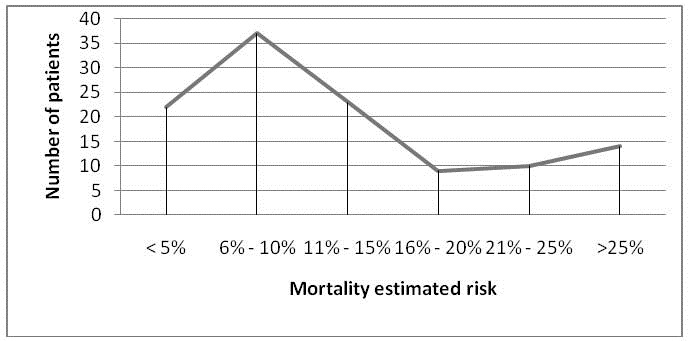
Mortality estimated risk

The estimated mortality risk in our group based on POSSUM score was the following: less than 5% - 22 patients (19%); risk between 6% and 10% - 37 patients (32%); risk 11% to 15% - 23 patients (20%); 16% to 20% mortality risk – 9 patients (7,8%); 21% to 25% - 10 patients (8,7%); over 25% risk – 14 patients (12%) (Fig. 4)

The average estimated risk of mortality for the entire group was 12.73%.

The number of deaths observed in our group of patients was 52, representing 45.21%. This results in a report of cases observed / expected at 3.55 / 1 for the whole group.

The bundling patients by Child evolutionary stages led to the observation that the reporting observed to the estimated results based on POSSUM score figure varies from one group to another (Table 3).

**Table 3:**
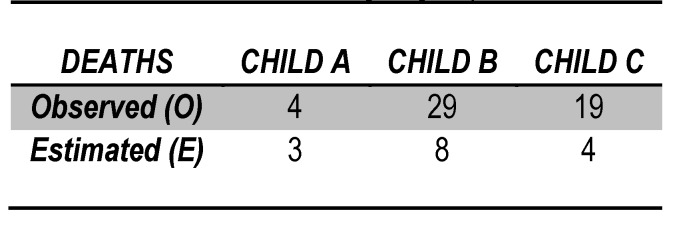
POSSUM score according to groups

The group of patients in stage Child A of liver cirrhosis had a morbidity of 48.5% (16 cases) and a mortality rate of 9.7% (4 cases).

Based on POSSUM score estimation, morbidity in these patients would have a percentage of 42.54 and a mortality rate of 9.1%, values which are quite similar to those registered by us.

The ratio O / E for patients with liver cirrhosis in stage Child A was 1.1 for morbidity and 1,2 for mortality. 

The null hypothesis was that the evolutionary stages of liver cirrhosis had no influence on POSSUM score prediction ability.

The statistical analysis based on X² test ("goodness of fit") for mortality occurred in patients belonging to Child class A, which resulted in the following value:

x² =(1, n=33) =0.36, p<0,05

The critical value for X2 is 3.84, placing the result in the acceptance area.

 Regarding the capacity of POSSUM score to estimate morbidity for our patients in stage Child A of liver disease, the statistical analysis based on X² test resulted in the following value:

x²=(1 , n=33)=0.35, p<0.05

Consequently, the predictive value of POSSUM scores in patients with liver cirrhosis in stage Child A was satisfactory both in terms of morbidity and mortality.

We found an exponential increase in the mortality rate – 29 deaths from 54 – for patients in Child B stage of liver cirrhosis representing 53,7%.

The estimated rate of mortality based on POSSUM score for this group of patients was of 14% - 8 deaths. Thus, the observed/estimated ratio was 3,8/1 and the value for x²=(1, n=54)=64,7, p<0,05, placing the result in the rejection area. 

Of the 25 patients of Child B group who were discharged, 22 had postoperative complications.

Patients in stage Child C of liver disease had the highest death rates – 19 from a total of 28, representing 67,85%.

Compared to a total of 4 deaths expected in this group based on POSSUM score prediction the ratio O/E is 4,75 and the value for X² in rejection area.

All 9 patients belonging to Child C stage who survived surgery had postoperative complications requiring prolonged hospitalization.

## Discussion

Surgery for acute abdomen in a patient with liver cirrhosis is an unavoidable risk, but its quantification is particularly significant given that modern medicine is based on analysis, objectivity and rigorous control of medical care quality.

The outcome of surgery depends not only on the skills of the operator, but is the result of several factors such as the physiological status of the patient, the condition requiring surgery, type of operation, but also the resources for preoperative and postoperative resuscitation which the hospital unit can provide.

Operative risk in a patient with cirrhosis can be approximated in a wide range on the evolutionary stage of the liver disease [**13**,**16**]. For the final result, the magnitude of the surgery and the patient's condition at the time of intervention are important factors to consider. POSSUM score has a satisfying predictive value quantifying and integrating in its equations both the physiological parameters and the magnitude of the procedure.

Cirrhotic patients undergoing abdominal surgery are at a high risk of hepatic decompensation due to reduced hepatic blood flow during such interventions [**17**,**18**].

Considering all these elements in a 5-year study on a group of 115 patients hospitalized and operated in emergency for various conditions that can be defined as "acute abdomen" we assessed the grade of influence of underlying liver disease, physiological status and amplitude of surgical procedure on the postoperative outcome.

The data obtained in our study showed that when there is a functional satisfactory hepatic reserve, as in stage Child A of liver cirrhosis, the patient can tolerate the surgical act satisfactorily. At this stage of liver disease, patients can fit in the same category with the other patients in terms of predictive ability of POSSUM score.

For the advanced stages of cirrhosis the high rates of mortality brought out the results from the prediction area provided by the POSSUM score. It suggests that patients in stages Child B and C of liver cirrhosis, emergency abdominal surgery has a major impact on the homeostasis and the resources for compensation are overloaded. Thus, in advanced stages, the liver disease has a prominent value for prognosis as against the POSSUM score.

## Conclusions

Surgery for acute abdomen in patients with liver cirrhosis is a therapeutic challenge and for the surgeon it means taking an inevitably risk. The outcome of the surgical procedure is a resultant of multiple factors and some of them can be quantified and integrated in valuable predictive scores such as POSSUM.

 According to our study, POSSUM score offers a satisfactory prediction for morbidity and mortality in emergency abdominal surgery for patients in compensated stages of liver cirrhosis – Child A. For the advanced cirrhosis stages, Child B and C, high levels of mortality cannot be predicted by using POSSUM score and liver disease has a prominent value in prognosis of this category of patients.
